# Gsα deficiency facilitates cardiac remodeling via CREB/ Bmp10-mediated signaling

**DOI:** 10.1038/s41420-021-00788-3

**Published:** 2021-12-14

**Authors:** Ping Yin, Dan Li, Qi Zhao, Mingming Cai, Zhenru Wu, Yujun Shi, Li Su

**Affiliations:** 1 Department of Cardiology, the Second Affiliated Hospital of Chongqing Medical University, Chongqing, 400010 China; 2 Laboratory of Pathology, West China Hospital, Sichuan University, Chengdu, Sichuan 610041 China

**Keywords:** Heart failure, Cardiovascular genetics

## Abstract

The stimulatory G-protein alpha subunit (Gsα), a ubiquitously expressed protein, mediates G-protein receptor-stimulated signal transduction. To investigate the functions of Gsα in cardiomyocytes. We developed transverse aortic constriction (TAC)-induced heart failure mouse models and tamoxifen-inducible transgenic mice with cardiac-specific Gsα disruption. We detected alterations in Gsα expression in TAC-induced heart failure mice. Moreover, we examined cardiac function and structure in mice with genetic Gsα deletion and investigated the underlying molecular mechanisms of Gsα function. We found that Gsα expression increased during the compensated cardiac hypertrophy period and decreased during the heart failure period. Moreover, cardiac-specific Gsα disruption deteriorated cardiac function and induced severe cardiac remodeling. Mechanistically, Gsα disruption decreased CREB1 expression and inhibited the Bmp10-mediated signaling pathway. In addition, we found that Gsα regulates Bmp10 expression through the binding of CREB1 to the Bmp10 promoter. Our results suggest that fluctuations in Gsα levels may play a vital role in the development of heart failure and that loss of Gsα function facilitates cardiac remodeling.

## Introduction

Heart failure (HF) is a complex clinical syndrome characterized by the impairment of ventricular filling or ejection [1]. HF has high global mortality and morbidity rates. It is expected to affect more than 8 million people aged ≥18 years in the United States by 2030 [2, 3]. Although various internal or external pathogenic stimuli cause HF, adverse cardiac remodeling, which is own to the over-activation of fibroblasts and excessive deposition of extracellular matrix caused by cardiomyocytes death, is the common and vital pathological basis for the disease [4]. Cardiac remodeling is a prognostic indicator of clinical HF and a key therapeutic target in patients with HF [5]. Therefore, understanding the mechanisms of cardiac remodeling and identifying effective therapeutic targets is crucial for the treatment of HF.

The stimulatory G-protein alpha subunit (Gsα) is a ubiquitously expressed protein, encoded by GNAS (*GNAS* in humans and *Gnas* in mice); it couples with seven transmembrane receptors for signal transduction by inducing adenylate cyclase (AC) to generate cyclic adenosine monophosphate (cAMP), which in turn activates the protein kinase A (PKA) [6]. PKA phosphorylates various downstream substrates, including the cAMP response element-binding protein (CREB), at Ser-133. CREB then binds to target genes and regulates their expression [7, 8]. Many studies have found that mutations in *GNAS* are associated with human diseases; *GNAS*-activating mutations lead to McCune-Albright syndrome, while *GNAS*-inactivating mutations cause Albright’s hereditary osteodystrophy [9, 10]. Previous studies conducted on transgenic mice revealed that homozygous Gsα knockout (KO) mice died during the embryonic period. Although heterozygous Gsα KO mice survived, they ultimately developed distinct metabolic phenotypes owing to the genomic imprinting of *Gnas* [9, 11]. To circumvent these issues and evaluate the effect of Gsα deficiency in specific tissues, mice with Gsα exon1 floxed with loxp recombination were generated. Recent reports have shown that abnormal Gsα expression can perturb cell metabolism, growth, differentiation, proliferation, apoptosis, and contraction [12]. Smooth muscle-specific Gsα deficiency disables intestinal contractile function and promotes the formation of abnormal aortic aneurysms [13, 14]. Liver-specific Gsα deficiency inhibits glucose metabolism and impairs liver regeneration [12, 15]. However, the impact of Gsα deletion in cardiomyocytes has not been investigated.

In the present study, a pressure overload-induced HF mouse model was generated through a transverse aortic constriction (TAC) and used to explore Gsα expression during HF. Subsequently, Myh6-MerCreMer^+/−^/Gsα^flox/flox^ mice were generated to investigate the function of Gsα in cardiomyocytes.

## Results

### Gsα expression is upregulated in the compensatory hypertrophic stage and downregulated in the heart failure stage

**Fig. 1:**
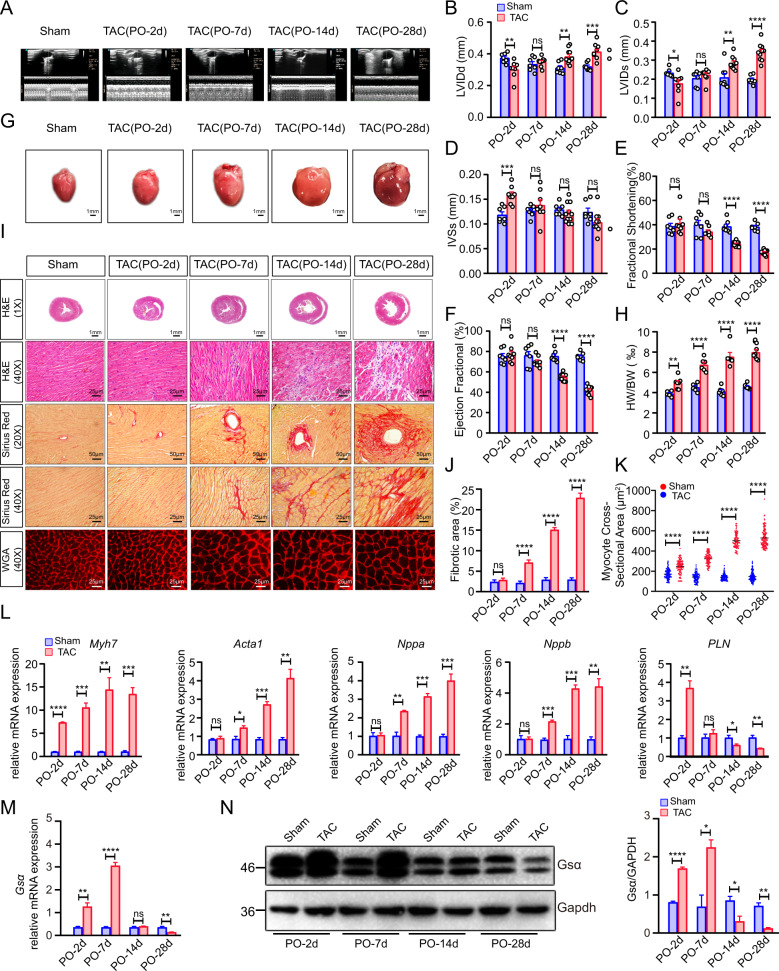
Gsα expression is upregulated in the compensatory hypertrophic stage and downregulated in the heart failure stage. **A** Representative raw images of M-mode echocardiography. **B–F**: Cardiac function parameters of Left ventricular internal diameters at diastole (LVIDd, mm) (**B**); Left ventricular internal diameters at systole (LVIDs, mm) (**C**); interventricular septal thickness at systole (IVSs, mm) (**D**); Fractional shortening (FS, %) (**E**); Ejection fraction (EF, %), *n* = 7–10 per group. **G** Gross morphology of heats. **H** Quantitative analysis of the ratio of heart weight to body weight (HW/BW, ‰), *n* = 5–9 per group. **I** Histopathological analysis on paraffin sections with hematoxylin-eosin (H&E) staining(1**×**, scale bar = 1 mm; 40**×**, scale bar = 25 μm), Sirius Red staining(20**×**,scale bar = 50 μm; 40**×**,scale bar = 25 μm), and Wheat germ agglutinin(WGA) staining (scale bar = 25 μm). **J** Quantitative analysis of the fibrotic area (%), *n* = 3 per group. **K** Quantitative analysis of the myocyte cross-sectional area (μm^2^). **L** Quantitative analysis of the mRNA expression of Myh7, Acta1, Nppa, Nppb, and PLN, *n* = 3 per group. **M** Quantitative analysis of the mRNA expression of Gsα, *n* = 3 per group. **N** Western blot analysis of Gsα protein expression, GAPDH was used as a loading control. Mean ± SEM, *p < 0.05, **p < 0.01, ***p < 0.001, ****p < 0.0001 vs Sham group. Statistical analysis was carried out by a two-tailed Student’s *t*-test.

To explore Gsα expression during HF, TAC was used to generate pressure overload-induced heart failure, mouse models. Echocardiographic analysis (Fig. 1A and Supplementary Fig. 1A) showed that, compared to mice in the Sham group, mice in the TAC group exhibited a significant decrease in left ventricular internal diameter at end-diastole (LVIDd) (Fig. 1B) and left ventricular internal diameter at end-systole (LVIDs) (Fig. 1C), as well as a significant increase in interventricular septum end-systolic thickness (IVSs) (Fig. 1D) on day 2 post-operation (PO-2d). However, mice in the TAC group gradually developed dilated ventricular chambers, as reflected by their significantly increased LVIDd, LVIDs, and slightly reduced IVSs, at later time points (PO-14d and PO-28d) (Fig. 1B–D). Cardiac systolic functioning in the TAC group, as evidenced by the left ventricular ejection fraction (LVEF) and fractional shortening (FS), was similar to that in the Sham group at early time points (PO-2d and PO-7d) (Fig. 1E, F), but gradually decreased at later time points, reaching levels of 30 and 25%, respectively, on PO-28d (Fig. 1E, F). Necropsy findings revealed that mice in the TAC group exhibited a sustainable increase in heart size (Fig. 1G), which was reflected by a persistent increase in the heart weight to body weight ratio (Fig. 1H).

Histological analysis of paraffin-embedded sections stained with hematoxylin and eosin(H&E), Sirius red, and wheat germ agglutinin (WGA) showed concentric hypertrophy, a slight degree of inflammatory infiltration, and myocyte hypertrophy, in the TAC group at early time points, but no additional cardiomyocyte disorders nor cardiac fibrosis, (Fig. 1I–K); however, eccentric hypertrophy, significant inflammatory infiltration, myocyte hypertrophy, severe perivascular fibrosis, and interstitial fibrosis were observed in the TAC group at later time points (Fig. 1I–K). Consistent with the above findings, measurement of cardiac hypertrophy marker levels using quantitative reverse transcription-polymerase chain reaction(RT-qPCR) revealed a persistent increase in myosin heavy chain 7(*Myh7*) levels by PO-2d and a significant increase in natriuretic peptide a (*Nppa*), actin alpha 1 (*Acta1*), and natriuretic peptide b (*Nppb*) levels by PO-7d, while a decrease in Phospholamban (*PLN*) levels from PO-7d, indicating the onset of decompensated cardiac hypertrophy (Fig. 1L).

Unlike that in the Sham group, Gsα mRNA expression markedly increased in the TAC group at early time points but progressively decreased at late time points (Fig. 1M). A similar trend was observed for Gsα protein expression in the TAC group (Fig. 1N).

In summary, these results indicate that variations in Gsα expression were closely associated with the progression of TAC-induced HF and that Gsα may play a vital role in HF

### Cardiac-specific Gsα deletion in adult mouse hearts decreases cardiac function

**Fig. 2:**
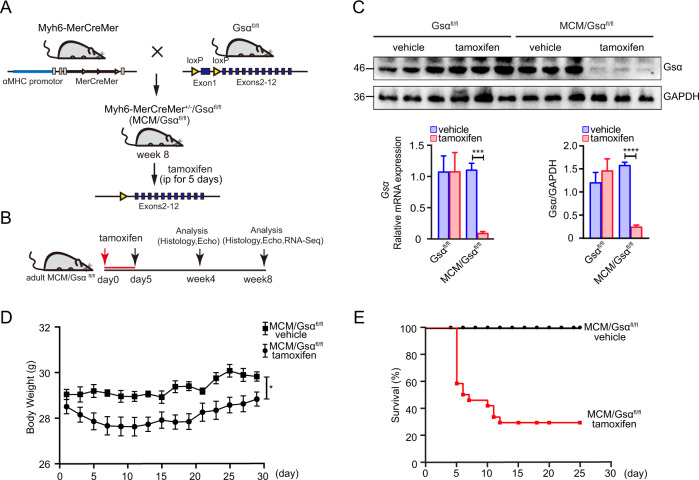
Efficient deletion of Gsα in adult mice. **A** Schematic diagram of generating Gsα^CMKO^ mice induced by tamoxifen treatment. **B** Flow diagram of experiment. **C** RT-qPCR and Western blot analysis for Gsα knockout efficiency. **D** Growth curve of control mice and Gsα^CMKO^ mice after tamoxifen treatment. **E** Kaplan–Meier survival plot of control mice and Gsα^CMKO^ mice after tamoxifen treatment, *n* = 100 Mean ± SEM, *p < 0.05, ***p < 0.001, ****p < 0.0001 vs control group. Statistical analysis was carried out by a two-tailed Student’s *t*-test.

To explore the function of Gsα in cardiomyocytes in vivo, Gsα^flox/flox^ mice were crossed with tamoxifen-inducible Myh6-MerCreMer transgenic mice to generate Myh6-MerCreMer^+/−^/Gsα^flox/flox^ (MCM/Gsα^fl/fl^) mice. To induce cardiac-specific deletion of Gsα (Gsα^CMKO^), adult male MCM/Gsα^fl/fl^ mice were administered tamoxifen for 5 consecutive days (Fig. 2A, B). In addition, MCM/Gsα^fl/fl^ mice were administered the vehicle, and Gsα^fl/fl^ mice were administered tamoxifen or the vehicle, were used as controls. As a confirmation of the efficiency of Gsα deletion, 4 weeks after tamoxifen treatment, cardiac Gsα mRNA and protein levels were almost undetectable in Gsα^CMKO^ mice, as determined via RT-qPCR and western blotting. (Fig. 2C). Moreover, 1 week after tamoxifen treatment, the Gsα^CMKO^ mice showed reduced body weights compared to mice in the control group (Fig. 2D) and the Gsα^CMKO^ mice exhibited reduced activity and reduced food consumption. In consonance with the above findings, up to 60% of Gsα^CMKO^ mice died within 7 days of tamoxifen treatment, and the mortality rate rose to ~70% in the 2 weeks following the treatment (Fig. 2E).

**Fig. 3:**
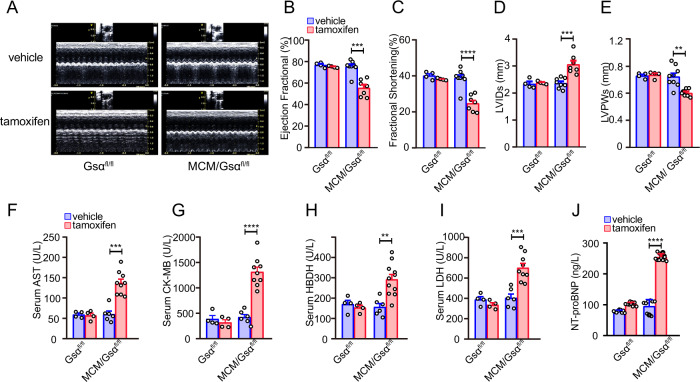
Cardiac-specific deletion of Gsα in adult mice provoke cardiac dysfunction. **A** Representative raw images of M-mode echocardiography. **B–E** Cardiac function parameters of ejection fraction (EF, %) (**B**); fractional shortening (FS, %) (**C**); left ventricular internal diameters at systole (LVIDs, mm) (**D**); left ventricular posterior wall thickness at systole (LVPWs, mm) (**E**), *n* = 4–9 per group. **F**–**I** Serum levels of aspartate aminotransferase (AST) (**F**), creatine kinase (CK) (**G**), lactic dehydrogenase (LDH) (**H**), and α-hydroxybutyrate dehydrogenase(α-HBDH) (**I**) were measured in Gsα^CMKO^ mice and control mice at 8 weeks after tamoxifen or vehicle treatment. *n* = 4–9 per group. **J** Serum levels of NT-proBNP were measured in Gsα^CMKO^ mice and control mice by ELISA. *n* = 9 per group. Mean ± SEM, **p < 0.01, ***p < 0.001, ****p < 0.0001 vs control group. Statistical analysis was carried out by a two-tailed Student’s *t*-test.

To evaluate the impact of Gsα deficiency on cardiac function, 8 weeks after tamoxifen or vehicle treatment, the cardiac functioning of Gsα^CMKO^ mice and mice in the control groups was evaluated through non-invasive echocardiography (Fig. 3A and Supplementary Fig. 1B). As expected, the vehicle-treated Gsα^fl/fl^ mice, MCM/Gsα^fl/fl^ mice, and tamoxifen-treated Gsα^fl/fl^ mice showed no changes in EF or FS, or in any other indices. However, Gsα^CMKO^ mice exhibited deteriorated cardiac function, as indicated by the significant decreases in LVEF and FS of ~40% (Fig. 3B) and 25%, respectively (Fig. 3C). In addition, the Gsα^CMKO^ mice exhibited dilated left ventricles, as confirmed by an ~30% increase in LVIDs (Fig. 3D) and an ~20% decrease in left ventricular posterior wall thickness at end-systole (LVPWs) (Fig. 3E). Furthermore, we measured serum heart injury and failure biomarker levels at the same time point, including aspartate aminotransferase (AST), creatine kinase-MB (CK-MB), lactic dehydrogenase (LDH), α-hydroxybutyrate dehydrogenase (α-HBDH), and n-terminal pro-brain natriuretic peptide(NT-proBNP), and found that the levels of the five biomarkers were significantly increased in Gsα^CMKO^ mice, compared to those of the mice in the control groups (Fig. 3F–J). In summary, cardiac-specific Gsα disruption was found to induce cardiac dysfunction in adult mice.

### Cardiac-specific Gsα deficiency in adult mice induces severe cardiac remodeling

**Fig. 4:**
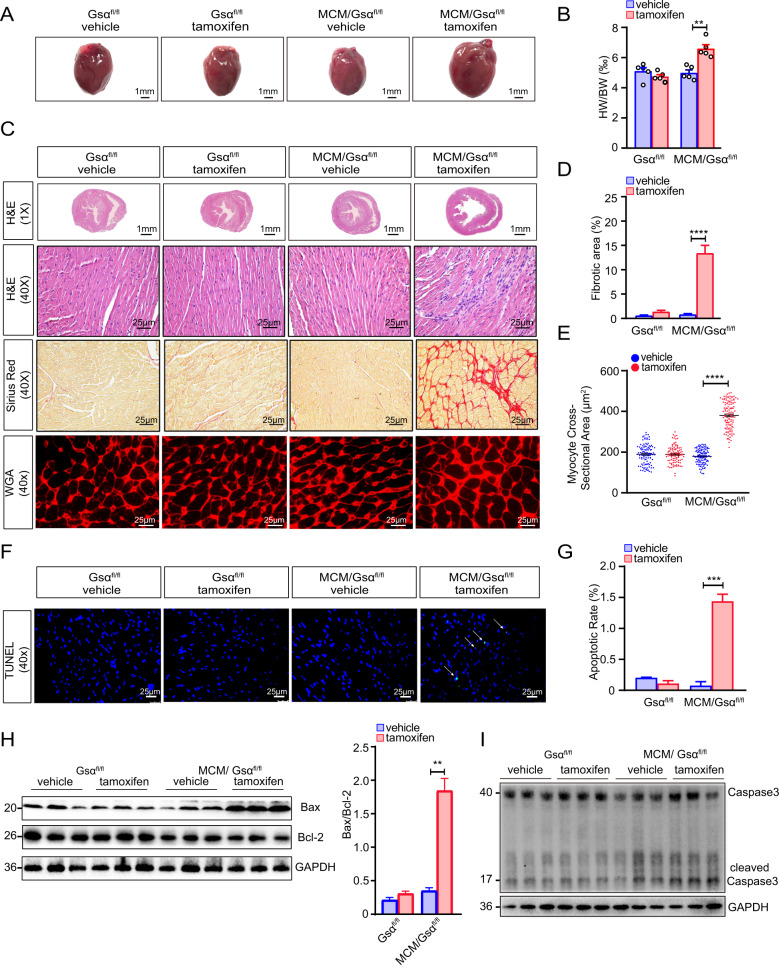
Cardiac-specific Gsα deficiency in adult mice confers severe cardiac remodeling. **A** Gross morphology of heats(scale bar = 1 mm). **B** Quantitative analysis of the ratio of heart weight to body weight (HW/BW, ‰), *n* = 5 per group. **C** Histopathological analysis on paraffin sections with hematoxylin-eosin(H&E) staining(1**×**, scale bar = 1 mm; 40**×**, scale bar = 25 μm), Sirius Red staining (scale bar = 25 μm)and Wheat germ agglutinin(WGA) staining(scale bar = 25 μm). **D** Quantitative analysis of the fibrotic area (%), *n* = 3 per group. **E** Quantitative analysis of the myocyte cross-sectional area (μm^2^). **F** TUNEL staining to detect myocardial apoptosis(scale bar = 25 μm). **G** Quantitative analysis of myocardial apoptosis, *n* = 3 per group. **H** Western blot analysis of Bax and Bcl2 of apoptosis and quantitative analysis of the ratio of Bax to Bcl2. **I** Western blot analysis of caspase3 and cleaved caspase3. Mean ± SEM, *p < 0.05, **p < 0.01, ***p < 0.001, ****p < 0.0001 vs control group. Statistical analysis was carried out by a two-tailed Student’s *t*-test.

To investigate the potential reasons for the deterioration of cardiac function in Gsα^CMKO^ mice, mouse hearts were harvested and the pathology was examined after echocardiographic assessment. Gsα^CMKO^ mice exhibited an increase in heart size (Fig. 4A), confirmed by the significant increase in heart weight adjusted for body weight (Fig. 4B). Histopathological analysis revealed that the Gsα^CMKO^ mice exhibited severe pathological phenotype, with dilated ventricle, disarrayed myofibers infiltrated inflammatory cells by H&E staining (Fig. 4C), with significant interstitial fibrosis by Sirius red staining (Fig. 4C, D), and with significantly hypertrophied myocytes by WGA staining (Fig. 4C, E). in addition, a significant increase in myocardial apoptosis was observed in Gsα^CMKO^ mice by transferase-mediated dUTP nick end-labeled (TUNEL) staining (Fig. 4F, G and Supplementary Fig. 1D). Importantly, we analyzed key molecules involved in apoptosis and found that the levels of Bax and the ratio of Bax/Bcl2 were markedly increased (Fig. 4H), and found that the cleaved caspase3 was also significantly increased in Gsα^CMKO^ mice and (Fig. 4I).

Taken together, these results demonstrated that cardiac-specific Gsα deletion induces severe cardiac remodeling with excessive interstitial fibrosis and cardiomyocytes apoptosis in adult mice.

### Gsα deletion downregulates BMP-mediated signaling and cAMP/CREB1 signaling

**Fig. 5:**
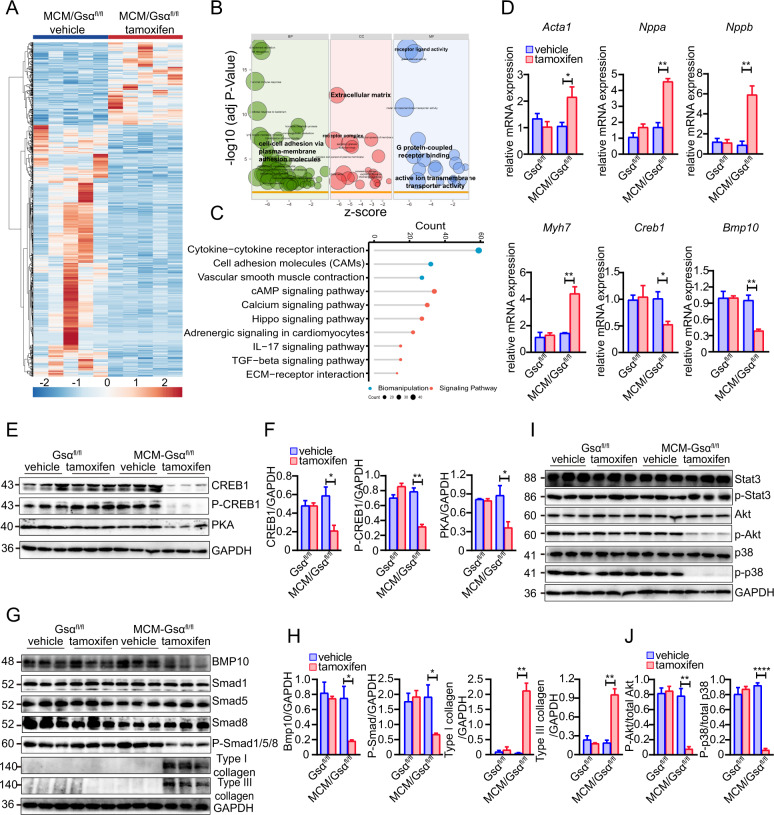
Gsα deletion downregulated BMP-mediated signaling and cAMP/CREB1 signaling. **A** Heat map representing differentially expressed genes between Gsα^CMKO^ mice and control mice **B** Gene ontology analysis of differentially expressed genes **C** KEGG analysis of top ten enriched pathways **D** Quantitative analysis of the mRNA expression of differentially expressed genes involving Acta1, Nppa, Nppb, Myh7, Creb1, and Bmp10, *n* = 3 per group **E** Western blot analysis of protein level of total CREB1, p-CREB1 (at ser-133), and PKA between Gsα^CMKO^ mice and control mice. **F** Quantitative analysis of the expression of CREB1/GAPDH, p-CREB1/GAPDH, and PKA/GAPDH, *n* = 3 per group **G** Western blot analysis of key signaling molecules of Bmp10-mediated Smad-dependent signaling at between Gsα^CMKO^ mice and control mice. **H** Quantitative analysis of the expression of Bmp10/GAPDH, p-Smad1/5/8 (at Ser463/Ser465/Ser467)/GAPDH, Type I collagen/GAPDH, and Type III collagen/GAPDH, *n* = 3 per group **I** Western blot analysis of key signaling molecules of Bmp10-mediated Smad-independent signaling between Gsα^CMKO^ mice and control mice. Including Stat3, p-Stat3 (at Tyr705), p-AKT (at Ser473), AKT, p-P38 (at Thr180/Tyr182), and P38. **J** Quantitative analysis of the expression of p-AKT/total AKT, p-P38/total P38, *n* = 3 per group. Mean ± SEM, *p < 0.05, **p < 0.01, ****p < 0.0001 vs control group. Statistical analysis was carried out by a two-tailed Student’s *t*-test.

To identify the mechanisms underlying the development of HF phenotypes in Gsα^CMKO^ mice, RNA-seq was performed in Gsα^CMKO^ and control mice to profile differentially expressed genes. Based on the criteria of fold change >2 or <0.5, and *p* < 0.05, a total of 1004 genes were found to be differentially expressed between the Gsα^CMKO^ and control mice, among which 248 and 756 were upregulated and downregulated, respectively (Fig. 5A). Gene ontology (GO) analysis revealed that Gsα was mainly involved in the regulation of cell adhesion among the biological processes, extracellular matrix among the cellular components, and membrane-mediated signaling pathways among the molecular function (Fig. 5B). The Kyoto Encyclopedia of Genes and Genomes (KEGG) was further analyzed to determine the most affected pathways involving the upregulated and downregulated genes. We found that the TGFβ/Bmp and cAMP/CREB1 signaling pathways were among the ten most affected signaling pathways (Fig. 5C). RT-qPCR and western blotting were performed to confirm the differentially expressed genes. RT-qPCR analysis revealed that cardiac fetal genes including *Acta1*, *Myh7*, *Nppb*, and *Nppa*, which are associated with heart failure, were significantly increased in Gsα^CMKO^ mice (Fig. 5D). Given Gsα is responsible for stimulating cAMP, activating PKA, and subsequently activating CREB1, thus PKA and CREB1 protein levels were measured. As expected, western blotting analysis revealed that the levels of both total CREB1 and phospho-CREB1 (at Ser-133) levels, as well as the level of PKA, were significantly reduced in Gsα^CMKO^ mice (Fig. 5E, F).

Numerous studies have demonstrated that TGFβ/Bmp signaling is involved in cardiac remodeling and HF [16–18], and have been determined to be the most affected pathways in Gsα^CMKO^ mice. *Bmp10*, a peptide growth factor, belonging to the TGFβ superfamily [19], is a differentially expressed gene that was found, through RNA-seq analysis, to be downregulated >threefolds. Consistent with that, both Bmp10 mRNA and protein levels were also significantly decreased in Gsα^CMKO^ mice (Fig. 5D, G). Given that Bmp10 elicits its biological function by activating Smad1/5/8 and other Smad-independent signaling pathways, such as TAK1/MEKK1, STAT3, and Ras [20], we measured the levels of key downstream molecules involved in Bmp10-meditated Smad-dependent and Smad-independent signaling pathways. Western blot analysis revealed that, compared to control mice, phospho-Smad1/5/8 was significantly downregulated in Gsα^CMKO^ mice (Fig. 5G, H); however, there were no alterations in total Smad1, Smad5, and Smad8 levels in Gsα^CMKO^ mice (Fig. 5G). In line with the pathological phenotypes, type I and type III collagen were significantly upregulated in Gsα^CMKO^ mice, compared to control mice (Fig. 5G, H). In addition, phospho-p38 and phospho-Akt were downregulated in Gsα^CMKO^ mice, while the total Akt, total p38, total STAT3, and phospho-STAT3 levels remained unchanged (Fig. 5I, J), indicating that TAK1-MEK1 might be involved in Bmp10-mediated signaling. In summary, these data indicate that cardiac-specific Gsα deletion leads to the downregulation of cAMP/PKA/CREB and Bmp10-mediated signaling, ultimately resulting in HF with cardiac dysfunction and severe cardiac remodeling.

### rhBMP10 ameliorates cardiac dysfunction and cardiac remodeling in Gsα^CMKO^ mice

**Fig. 6:**
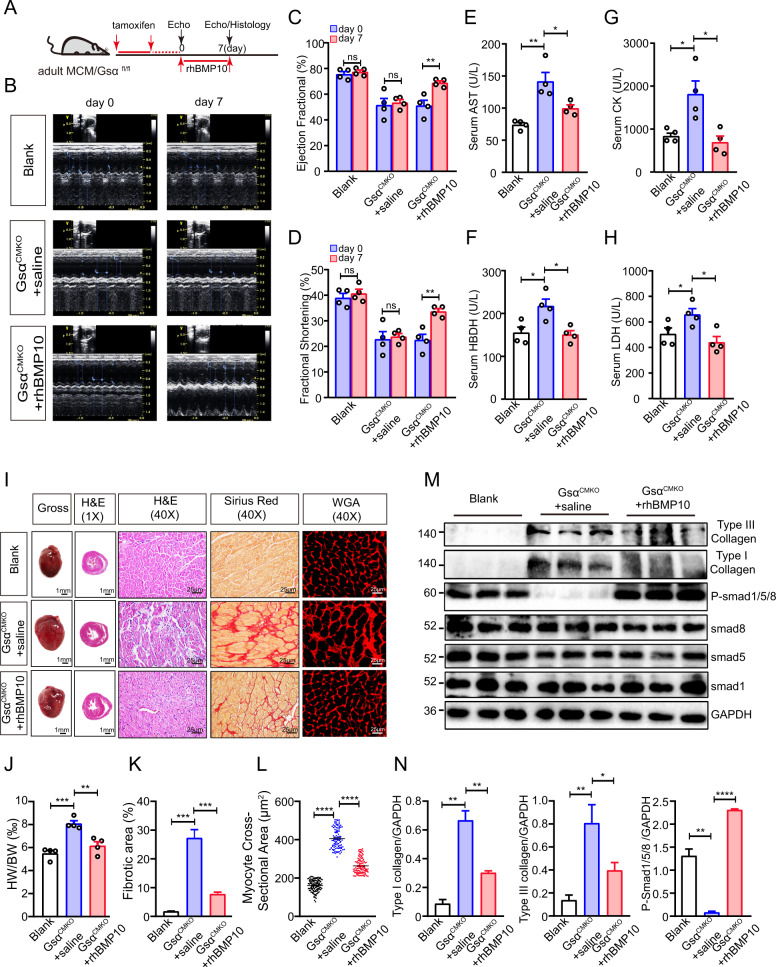
rhBMP10 ameliorate cardiac dysfunction and cardiac remodeling in Gsα^CMKO^ mice. **A** Flow diagram of the experiment. **B** Representative raw images of M-mode echocardiography. **C**, **D** Cardiac function parameters of ejection fraction (EF, %) (**C**) and fractional shortening (FS, %) (**D**). *n* = 4 per group. **E–H** Serum levels of aspartate aminotransferase (AST) (**E**), creatine kinase (CK) (**F**), α-hydroxybutyrate dehydrogenase (α-HBDH) (**G**), and lactic dehydrogenase (LDH) (**H**) were measured in all groups, *n* = 4 per group. **I** Gross morphology of heats (scale bar = 1 mm) and histopathological analysis with hematoxylin-eosin (H&E) staining (1**×**, scale bar =1 mm; 40**×**, scale bar = 25 μm), Sirius Red staining (scale bar = 25 μm), and Wheat germ agglutinin(WGA) staining(scale bar = 25 μm). **J** Quantitative analysis of the ratio of heart weight to body weight (HW/BW, ‰), *n* = 4 per group. **K** Quantitative analysis of the fibrotic area (%), *n* = 3 per group. **L** Quantitative analysis of the myocyte cross-sectional area (μm^2^). **M** Western blot analysis of key molecules of Bmp10-mediated Smad-dependent signaling. **N** Quantitative analysis of the expression of Type I collagen/GAPDH, Type III collagen/GAPDH, and p-Smad1/5/8 (at Ser463/Ser465/Ser467)/GAPDH. *n* = 3 per group. Mean ± SEM, *p < 0.05, *p < 0.05, **p < 0.01, ***p < 0.001, ****p < 0.0001. Statistical analysis was carried out by a two-tailed Student’s *t*-test.

To further confirm whether the development of the HF phenotype in Gsα^CMKO^ mice is dependent on Bmp10-mediated signaling, recombinant human Bmp10 (rhBmp10) was evaluated in Gsα^CMKO^ mice. In detail, Adult MCM/Gsα^fl/fl^ mice were intraperitoneally injected with tamoxifen or the vehicle for 7 consecutive days. After 15 days of recovery, the Gsα^CMKO^ mice were intraperitoneally injected with rhBmp10 or saline for 7 consecutive days (Fig. 6A, Supplementary Fig. 1C), while the vehicle-treated MCM/Gsα^fl/fl^ mice were intraperitoneally injected with only saline for 7 consecutive days (Blank group). Echocardiography was performed on days 0 (the first day of rhBMP10 treatment)and day 7 (the 7th day after rhBMP10 treatment) (Fig. 6B). As expected, Gsα^CMKO^ mice exhibited significant dysfunction after tamoxifen treatment for consecutive 5 days, while no significant change in EF and FS in Gsα^CMKO^ mice were found following the saline treatment for 7 days. In contrast, Gsα^CMKO^ mice treated with rhBMP10 showed significant increases in EF and FS of more than 40% and 50%, respectively (Fig. 6C, D). Serum AST, LDH, CK, and a-HBDH levels in Gsα^CMKO^ mice treated with rhBMP10 were significantly lower than those in Gsα^CMKO^ mice treated with saline but higher than those in the blank group (Fig. 6E–H). Heart tissues were harvested at the end of the experiment. The hearts of Gsα^CMKO^ mice treated with rhBMP10 were smaller than those of Gsα^CMKO^ mice treated with saline; this was reflected by the decrease in the heart weight adjusted to body weight value (Fig. 6I, J). As expected, H&E and Sirius red staining analysis revealed that Gsα^CMKO^ mice treated with saline exhibited severe cardiac remodeling, with dilated ventricles, myocyte disarray, significant inflammatory infiltration, significant interstitial fibrosis, and hypertrophied myocytes. However, treatment of Gsα^CMKO^ mice with rhBMP10 alleviated cardiomyocyte disorders, reduced myocardial fibrosis, and narrowed the myocyte cross-sectional area (Fig. 6I, K, L). As Bmp10 mainly exerts its biological function via Smad-dependent signaling, we observed that rhBMP10 infusion reduced type I and type III collagen protein levels, and increased p-Smad1/5/9 protein levels. In summary, these results suggest that the development of the HF phenotype in Gsα^CMKO^ mice was dependent on Bmp10-mediated signaling.

### CREB1 binds to the BMP10 promoter and regulates its expression

**Fig. 7:**
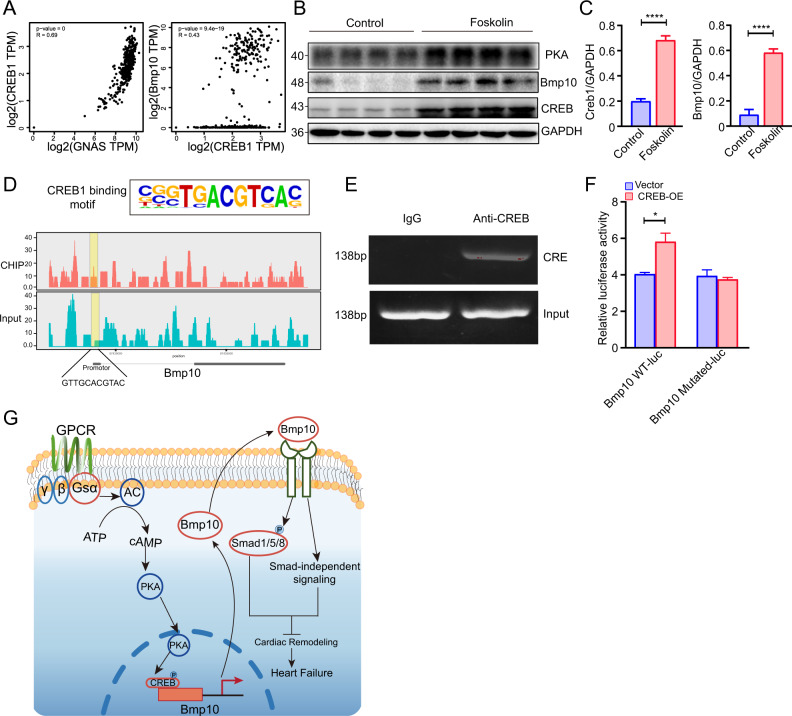
CREB1 binds with BMP10 promotor and regulates its expression. **A** Correlation of Gnas and CREB1, as well as the CREB1 and Bmp10 in the GTEx database. **B** Western blot analysis of CREB1 and Bmp10 on mice treated with forskolin for 2 days. *n* = 4 per group **C** Quantitative analysis of the expression of CREB1/GAPDH and Bmp10/GAPDH. *n* = 4 per group. **D** CREB1 binding motif in HOMER database and ChIP-Seq analysis of CREB1 binding site in Bmp10 promotor region. **E** ChIP-PCR analysis to verify the binding site of CREB1 in the Bmp10 promotor. **F** Luciferase reporter assay in 293 T cells. *n* = 3 per group **G** Schematic diagram of Gsα-regulated cardiac function and cardiac structure via CREB1/Bmp10-mediated signaling. Mean ± SEM, *p < 0.05, ****p < 0.0001. Statistical analysis was carried out by a two-tailed Student’s *t*-test.

Previous studies have shown that the transcription factor, CREB1, is involved in the pathophysiology of cardiovascular diseases [7, 21]. Based on data from the Genotype-Tissue Expression (GTEx) database, we found a significant correlation between GNAS and CREB1, as well as between CREB1 and Bmp10 (Fig. 7A). Subsequently, forskolin, an adenylate cyclase agonist, was intraperitoneally injected in wild–type (wt) mice at a dose of 0.5 mg/kg for 2 consecutive days. Mice treated with DMSO were used as the control. Western blot analysis revealed that forskolin indeed induced the expression of PKA as expected, and also the level of CREB and Bmp10 in mouse hearts (Fig. 7B, C). We hypothesized that Gsα regulates Bmp10 expression via CREB1. Generally, CREB1 is thought to induce the expression of target genes by binding to the CREB-response element (CRE) within the promoter regions of target genes [22, 23]. To verify this assumption, the transcription factor database (http://jaspar.generge.net) was used to predict potential CRE sites within the proximal 2 kb promoter region of Bmp10. In addition, chromatin immunoprecipitation followed by sequencing (ChIP-Seq) was performed on heart tissues of wt mice to determine the target genes of CREB1. Analysis of the ChIP-Seq data revealed that the CREB binding motif was located in the enrichment peaks within the Bmp10 promoter region (Fig. 7D). Furthermore, a polymerase chain reaction (PCR) was performed to verify whether CREB1 directly binds to Bmp10 (Fig. 7E). Moreover, we cloned the Bmp10 promoter region-luciferase reporter plasmid. The luciferase assay revealed that CREB1 overexpression significantly increased luciferase activity in the Bmp10 wt-luc group, while the Bmp10 mutated-luc group showed no change in luciferase activity (Fig. 7F). In summary, CREB1 modulated Bmp10 expression by directly binding to the Bmp10 promoter.

## Discussion

In the present study, a pressure overload-induced HF model was generated. Gsα expression was found to increase during the compensated period and decrease during the HF period, suggesting that Gsα plays a pivotal role in the pathophysiology of HF. Inducible Myh6-MerCreMer^+/–^/Gsα^flox/flox^ mice were then generated to evaluate the effect of Gsα ablation in cardiomyocytes. Interestingly, Gsα^CMKO^ mice exhibited cardiac dysfunction and severe cardiac remodeling, which is a hallmark of HF. Regarding the molecular mechanism of HF development, the Gsα^CMKO^ mouse HF phenotype might be dependent on Bmp10-mediated signaling pathways, and Gsα was found to regulate Bmp10 expression via CREB1 (Fig. 7G). Our study evaluated the role of Gsα in HF and demonstrated the molecular mechanisms underlying Gsα-deficiency-induced HF, thus improving our understanding of the function of Gsα in cardiomyocytes.

Previous studies have shown that tamoxifen treatment might induce cardiotoxicity with transient cardiomyopathy in MerCreMer transgenic mice [24]. To eliminate the cardiotoxicity caused by Cre nuclear translocation,

Myh6-MerCreMer transgenic mice were also treated with tamoxifen for 5 consecutive days, and no change of survival rate and cardiac function was found in Myh6-MerCreMer transgenic mice. Therefore, acute heart failure was assumed to be the cause of death in the Gsα^CMKO^ mice, but more evidences are needed to confirm the assumption in further study.

Numerous studies have investigated the multiple functions of Gsα in cardiomyocytes using loss- or gain-of-function approaches. Young transgenic mice with cardiac-specific Gsα overexpression only exhibited an enhanced response to catecholamines but developed dilated cardiomyopathy as they aged [25–28]. Another study reported that the attenuation of Gsα expression led to bradycardia and protected against isoproterenol-induced hypertrophy [29]. However, in our study, Gsα^CMKO^ mice showed cardiac dysfunction and cardiac remodeling at a young age, and there were no significant differences in heart rate under anesthetic conditions between Gsα^CMKO^ and control mice. The distinct phenotype of Gsα^CMKO^ mice can be explained as follows; first, Gsα overexpression mimicked chronic sympathetic stimulation throughout the life of the animals and resulted in cardiomyopathy at an older age. In contrast, cardiac-specific Gsα deletion rapidly inhibited signal transduction and signaling pathways, ultimately leading to HF. Second, the different methods may be responsible. For example, in a previous study [29], Gsα expression was found to be attenuated through the systemic overexpression of its dominant-negative mutant; however, the Gsα deletion in our study was performed using tamoxifen-inducible Cre recombinase, which might have high and long-term efficiency. Future studies should provide in-depth information on the influence of genetic Gsα deletion over an extended period in Gsα^CMKO^ mice.

Although we explored the alternation of Gsα in the HF model induced by TAC and the phenotype of cardiac-specific Gsα deletion. The relationship between the Gsα deficiency and HF model induced by TAC hasn’t been illustrated. We hypothesized that there might be an accumulative effect among the Gsα deficiency and HF. Exploring the influence on the cardiac-specific Gsα^flox/–^ haploinsufficient mice is needed in our further studies.

Our study revealed that Gsα^CMKO^ mouse phenotype depends on the downregulation of Bmp10 and the signaling pathways it mediates. Bmp10, which belongs to the TGF-β superfamily, is enriched in the heart and plays a vital role in vascular remodeling and cardiac development [30–32]. Bmp10^–/–^ embryos were found to succumb to HF induced by severely hypoplastic hearts [19] and transgenic mice with overexpression of Bmp10 exhibited a 50% reduction in heart size [33]. In addition, Bmp10 overexpression has been reported to exert a cardioprotective effect in ISO-induced HF models [34]. In our study, we found that cardiac-specific Gsα deletion downregulated Bmp10-mediated signaling and consequently provoked cardiac remodeling and found that rhBMP10 might ameliorate the phenotype induced by cardiac-specific Gsα deletion. Moreover, ChIP-PCR and luciferase analysis revealed that Gsα downregulated the expression of Bmp10 through the direct binding of CREB1 to the Bmp10 promoter. However, whether the effect on HF of rhBMP10 is direct or indirect and whether there are some other cofactors that negatively effected the transcription of Bmp10 induced by CREB, We will investigate these assumptions in our further studies.

In summary, our results suggest that Gsα levels fluctuate during HF, and that genetic Gsα deletion leads to severe cardiac dysfunction and cardiac remodeling via CREB/Bmp10-mediated signaling. Therefore, alterations in Gsα expression are crucial in the development of HF.

## Materials and methods

### Animals

All mice were housed in a specific pathogen-free facility at a temperature of 24 °C, humidity level of 55 ± 5%, and a 12-h light/dark cycle. The mice had unlimited access to food and water. All animal experiments were approved by the Ethics Committee of Experimental Animal of West China Hospital of Sichuan University.

### Pressure overload-induced heart failure mouse models

Adult male C57BL/J wt mice (22–25 g) were randomly assigned to the TAC or Sham group. In brief, mice in the TAC group were anesthetized using 1.5% isoflurane and underwent a thoracotomy. The aortic arch was dissected free from the surrounding soft tissues and muscles. A 6-0 silk thread was passed under the aortic arch, and a blunted 28-gauge needle (0.35 mm) was placed around the aorta and removed immediately after ligation. The Sham group underwent the same procedure without aortic constriction. Cardiac function, histopathological and molecular analyses were performed on the mice on postoperative days PO-2, PO-7, PO-14, and PO-28.

### Generation of cardiac-specific Gsα KO mice

Homozygous Gsα^flox/flox^ mice were crossed with heterozygous Myh6-MerCreMer^+/–^ mice to generate inducible cardiac-specific Myh6-MerCreMer^+/–^/Gsα^flox/flox^ (*MCM*/Gsα^fl/fl^) mice. Genotypes were confirmed by PCR. The expression of Cre recombinase under the Myh6 promotor ensured cardiac specificity. To generate adult mice with cardiomyocyte-specific Gsα deletion (Gsα^CMKO^),eight-week-old male *MCM*/Gsα^fl/fl^ mice were intraperitoneally injected with tamoxifen (2 mg/day) for 5 consecutive days; while the control groups were comprised of *MCM*/Gsα^fl/fl^ mice treated with corn oil (vehicle) and Gsα^flox/flox^ littermates treated with tamoxifen or corn oil (vehicle).

### Regents and antibodies

Tamoxifen (54965-24-1) were purchased from Sigma-Aldrich (USA); Forskolin (HY-15371) was purchased from MedChemEexpress (USA). NT-proBNP measurement kit (JM-02841M1) was purchased from JINMEI BIOTECHNOLOGY (China). Recombinant human BMP10 (2926-BP-025) was purchased from R&D Systems (USA).

The following antibodies were used in Western Blot: phospho-creb (Cell Signaling Technology, 9197), phospho-Smad1/5/8 (Cell Signaling Technology, 13820), phospho-p38 MAPK (Cell Signaling Technology, 4511), phospho-Stat3 (Cell Signaling Technology, 9145), phospho-Akt (Cell Signaling Technology, 4060), SMAD1 (Proteintech, 10428-1-AP), SMAD5 (Proteintech, 12167-1-AP), SMAD9 (Proteintech, 16397-1-AP), AKT (Proteintech, 10176-2-AP), STAT3(Proteintech, 10253-2-AP), p38 (HUABIO, ET1702-65), BAX (Proteintech, 50599-2-Ig), Bcl2 (HUABIO, ET1702-53), cleaved Caspase3 (HUABIO, ET1608-64), BMP10 (HUABIO, ER65711), GNAS (Proteintech, 10150-2-AP), CREB (HUABIO, ET1601-15), PKA (HUABIO, ER64618), Collagen type I (Proteintech, 146-95-1-AP), Collagen type III (Proteintech, 22734-1-1-AP), and GAPDH (HUABIO, ET1601-4).

### Echocardiography

Mice were anesthetized using 1.5% isoflurane and placed in the supine position. Short-axis M-mode echocardiography, using a 13 MHz transducer (VIVIDi, General Electric Healthcare, I12L-RS, General Electric Healthcare), was performed on them to assess cardiac function indices. Diastolic function indices included LVIDd, IVSd, and LVPWd, while LVIDS, IVSs, LVPWs, LVEF, and FS.

### Histological analysis

Mouse hearts were harvested and fixed in 10% neutral formalin, embedded in paraffin, and serially sectioned at 4 μm. The sections were stained with hematoxylin and eosin (H&E) to assess cardiac morphology and with Sirius red to evaluate cardiac collagen content. Myocardial fibrosis was quantified using the ImageJ software (National Institutes of Health, Bethesda, MD, USA). Three fields were randomly selected for each sample, and each group included at least three independent samples. All histological analysis were implemented in a blinded manner.

### Immunofluorescence assay

Sections were either incubated with Trias Red-X conjugate of wheat germ agglutinin (WGA-Texas Red) (W21405, Invitrogen, USA) to visualize and measure the cardiomyocyte cross-sectional area or with TUNEL (KGA7071, KeyGEN Biotech, China) to assess myocardial apoptosis. The stained sections were visualized using a Leica DM4000B fluorescence microscope. For each sample, three fields were randomly selected to determine the apoptotic cell count and at least 100 cells with whole membranes were selected from each sample and evaluated using the ImageJ software (National Institutes of Health, Bethesda, MD, USA). Each group contained at least three independent samples.

### RNA isolation and reverse transcription-polymerase chain reaction

Total RNA was extracted from cardiac tissue using an RNA isolation kit (RE-03011, FORGENE, China) according to the manufacturer’s instructions. RNA was converted to cDNA using the HiScript III RT SuperMix (R323-01, Vazyme, China). The DNA template was then amplified using a Bio-Rad CFX96TM PCR system and a ChamQ^TM^ SYBR Color qPCR Master Mix (Q411-02/03, Vazyme, China). Relative gene expression was normalized to RPS11 using the 2^−△△CT^ method. The detailed sequences of the primers used in this study are listed in (Table 1).

**Table 1:** The primer sequences.

Gene	Forward primer	Reverse primer
Gnas	5′-GGTCTATCCGAGTGTACCCGA-3′	5′-GGCCTTCTCACTATCTCCGTTAAA-3′
Myh7	5′-GACTTCCGGCAGAGGTATCG-3′	5′-AGCCTCTCGGTCATCTCCTT-3′
Nppa	5′-CGGAGCCTACGAAGATCCAG-3′	5′-AAGCTGTTGCAGCCTAGTCC-3′
Nppb	5′-ATCTCAAGCTGCTTTGGGCA-3′	5′-ACAACAACTTCAGTGCGTTACAG-3′
ACTA1	5′-GAGCGTGGCTATTCCTTCGT-3′	5′-GAAACGCTCATTGCCGATGG-3′
PLN	5′-TACCTCACTCGCTCGGCTAT-3′	5′-ATGCAGATCAGCAGCAGACA-3′
CREB1	5′-AGGGCCTGCAGACATTAACC-3′	5′-AGCACTGCCACTCTGTTCTC-3′
Bmp10	5′-ATGGCTGAACTGCGGTTGTA-3′	5′-TTTTACGGTCCACGCCATCA-3′
RPS11	5′-AGATGAAGATGCAGAGGACCATT-3′	5′-GACGCTTCTCAAAGCGATTGT-3′

### Western blot analysis

Protein lysates were extracted from mouse heart tissues. Equal amounts of proteins were separated by sodium dodecyl sulfate-polyacrylamide gel electrophoresis and transferred to nitrocellulose membrane The membranes were blocked for 1 h using non-fat milk (5%) and incubated with primary antibodies overnight at 4 °C. The membranes were then incubated with horseradish peroxidase-conjugated secondary antibodies at room temperature for 1 h. Bands were visualized with a Bio-Rad chemiluminescence system using a chemiluminescent reagent (E411-03, Vazyme, China).

### Chromatin immunoprecipitation assay

The chromatin immunoprecipitation experiments were carried out under the instructions of Methods in Molecular Biology protocol [35]. Mouse hearts were harvested, washed with ice-cold PBS, and immediately cross-linked using 1% formaldehyde for 10 min at room temperature. The tissues were frozen in liquid nitrogen and ground with a lysis buffer to collect cell nuclei. A nucleus lysis buffer was added to the collected nuclei, and chromatin DNA was sheared to 200–500 bp fragments by sonication. The sonicated chromatin was incubated with rabbit IgG and anti-CREB1 antibodies. IgG was used as the negative control. The DNA libraries were sent for sequencing using the VAHTS Universal DNA Library PreP Kit for Illumina V3 (ND607-01, Vazyme, China). Primer sequences within the target gene promoter region were designed for PCR follows Bmp10, 5′-ATTGTGGAGTGTGCGTTGAC-3′ and 5′-GCCCAATCTACTGCTGATGC-3′. The PCR products were analyzed via agarose gel electrophoresis.

### Luciferase reporter assay

DNA fragments of the 2 kb Bmp10 promoter region and the mutant Bmp10 promoter region were transferred into a GV238 vector to generate wt-luc and mutated-luc plasmids, respectively. A mixture of the luciferase reporter plasmid, the CREB overexpression vector, and the Renilla plasmid was transfected into 293 T cells using an X-tremegene HP reagent (Roche, USA). After 48 h of incubation, cells were lysed and luciferase activity was evaluated using the Dual-Luciferase Assay System (Promega, USA).

### Bioinformatic analysis

The heat map was made via ClustVis web tools. The GO analysis and KEGG analysis was made by the R Studio software. The gene correlation were analyzed on GTEx via Gene Expression Profiling Interactive Analysis web tools. The ChIP analysis was performed by the SEQHEALTH Company (Wuhan, China)

### Statistical analysis

All results are presented as mean ± standard error of the mean (SEM). Data were analyzed using the GraphPad 8.0 statistical software (San Diego, USA). The Shapiro–Wilk test was used to evaluate the normality of the data distribution. For normally distributed data, the unpaired Student’s *t*-test (for comparing two groups) or one-way analysis of variance (ANOVA; for comparing multiple groups) was used to assess significance. For non-normally-distributed data, the Mann–Whitney *U*-test was used for analysis. Differences between groups were considered significant at *P* < 0.05.

## Supplementary information

Author Contributions Statement

supplement

## Data Availability

The datasets used during the study are available from the corresponding author on reasonable request.
