# Supplementary material for: Gsα deficiency facilitates cardiac remodeling via CREB/ Bmp10-mediated signaling
Source: Cell Death Discov. 2021 Dec 14;7:391. doi: 10.1038/s41420-021-00788-3 (PMC8671484; doi:10.1038/s41420-021-00788-3)
Supplement: Supplementary file 1 — Author Contributions Statement [file 41420_2021_788_MOESM1_ESM.docx]

**Author Contributions Statement**

**Gsα deficiency facilitates cardiac remodeling via CREB/ Bmp10-mediated signaling**

Ping Yin^1^, Dan Li^1^, Qi Zhao^1^, Mingming Cai^1^, Zhenru Wu^2^, Yujun Shi^2^, Li Su^1*^

1. *Department of Cardiology, the Second Affiliated Hospital of Chongqing Medical University, Chongqing 400010, China*
2. *Laboratory of Pathology, West China Hospital, Sichuan University, Chengdu, Sichuan 610041, China*

*** Corresponding author.**

**Ping Yin -Author 1 (First Author)**: Conceptualization, Methodology, Software, Investigation, Data Analysis, Plotting figures, Writing -Original Draft；

**Dan Li -Author 2:** Data Curation, Writing -Original Draft；

**Qi Zhao -Author 3:** Visualization, Investigation；

**Mingming Cai -Author 4:** Resources, Investigation；

**Zhenru Wu -Author 5:** Methodology；

**Yujun Shi -Author 6:** Visualization, Writing-Review & Editing；

**Li Su -Author 7 (Corresponding Author)**: Conceptualization, Funding Acquisition, Resources, Supervision, Writing -Review & Editing.
