# Supplementary material for: Gsα deficiency facilitates cardiac remodeling via CREB/ Bmp10-mediated signaling
Source: Cell Death Discov. 2021 Dec 14;7:391. doi: 10.1038/s41420-021-00788-3 (PMC8671484; doi:10.1038/s41420-021-00788-3)
Supplement: Supplementary file 2 — supplement [file 41420_2021_788_MOESM2_ESM.docx]

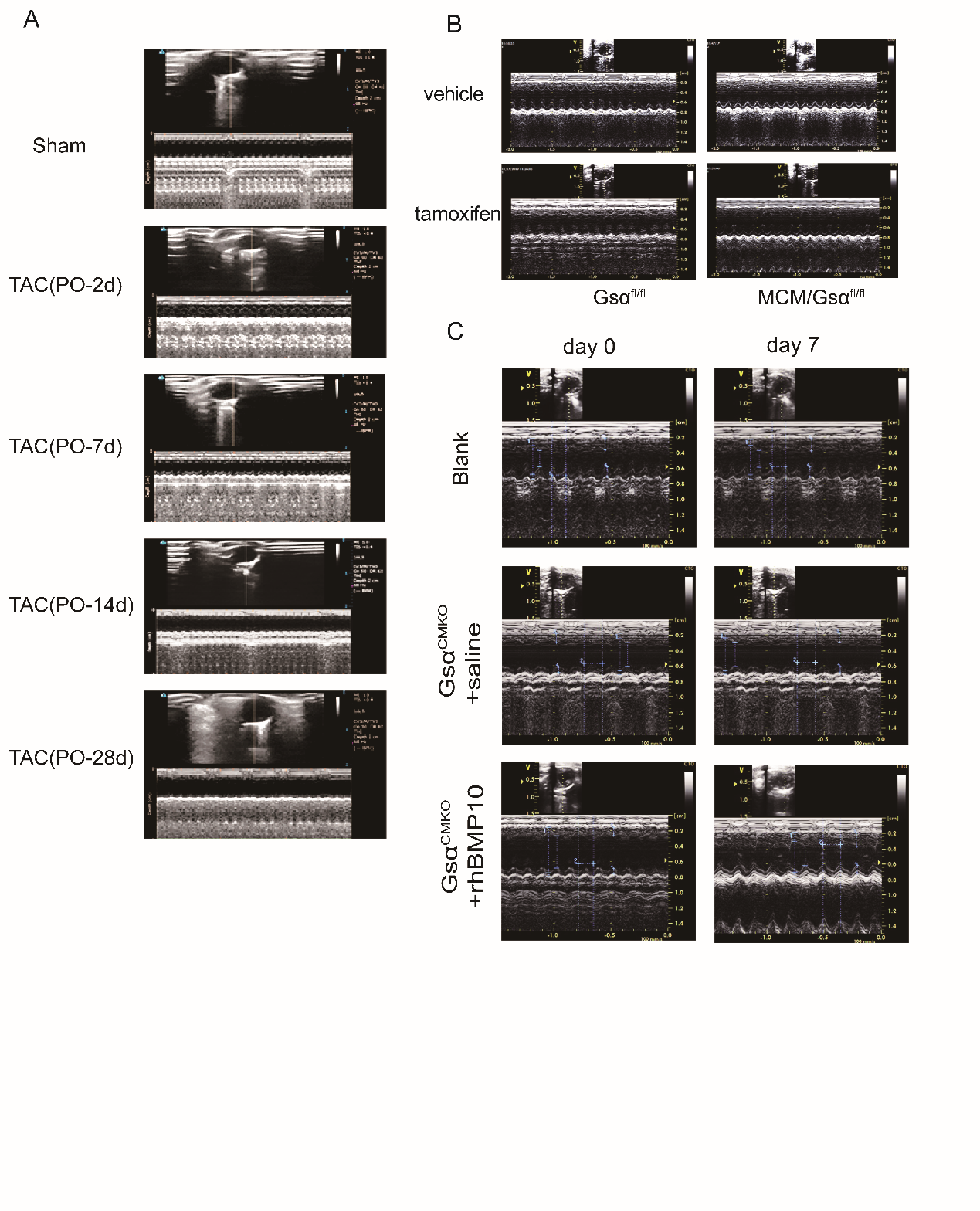


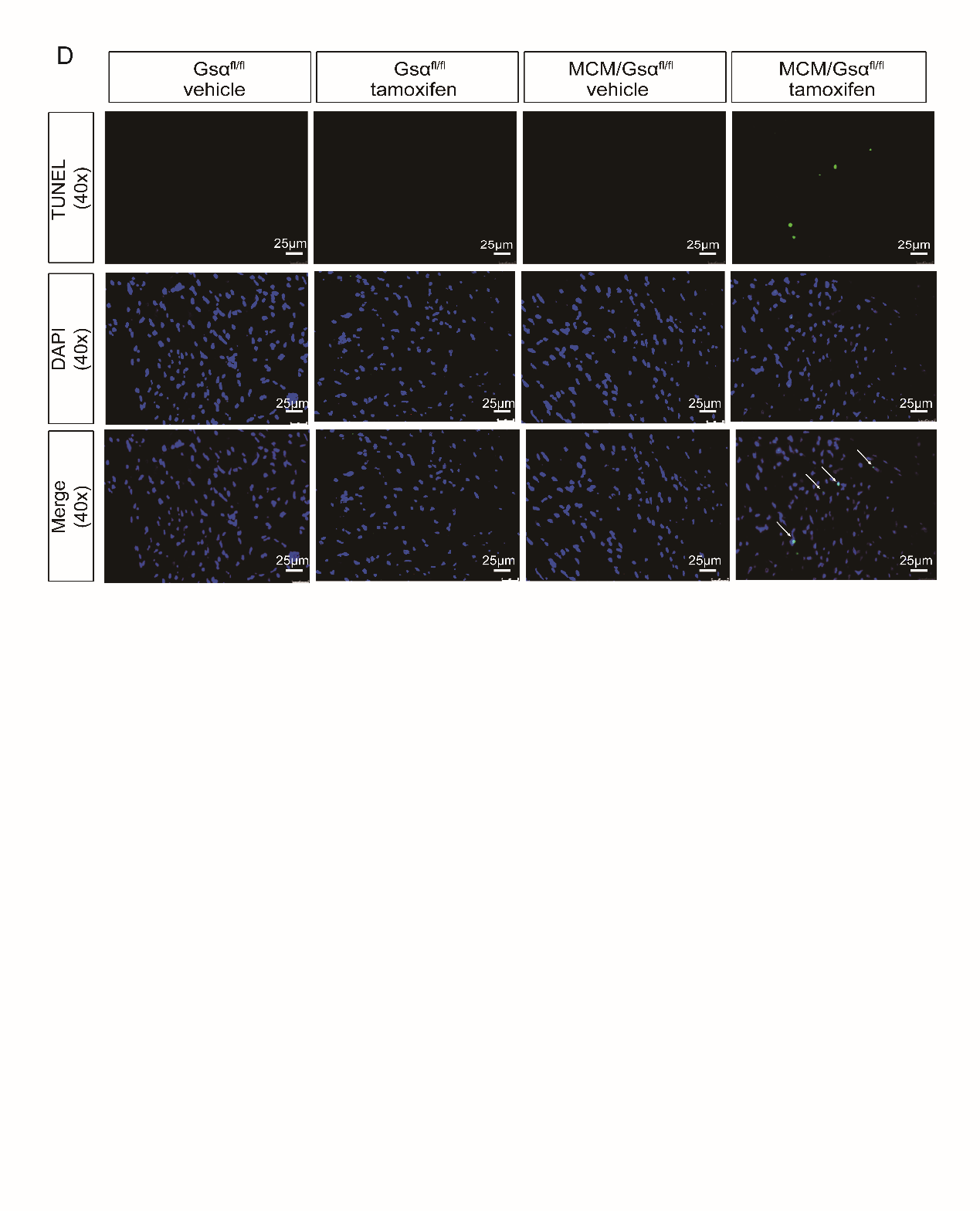


**Figure 1. A**: Enlarged representative raw images of M-mode echocardiography between TAC group and Sham group in different time point. **B.** Enlarged representative raw images of M-mode echocardiography between Gsα^CMKO^ mice and control mice. **C.** Enlarged representative raw images of M-mode echocardiography between Gsα^CMKO^ mice and control mice before and after using rhBMP10. **D.** images with TUNEL staining at the different emission wavelengths to detect myocardial apoptosis(scale bar=25 μm)
